# The Histone H3 Lysine 4 Presenter WDR5 as an Oncogenic Protein and Novel Epigenetic Target in Cancer

**DOI:** 10.3389/fonc.2018.00502

**Published:** 2018-11-14

**Authors:** Kebin Lu, He Tao, Xiaomin Si, Qingjuan Chen

**Affiliations:** ^1^Department of Paediatrics, Shan Xian Central Hospital, Heze, China; ^2^Department of Medical Oncology, Shan Xian Haijiya Hospital, Heze, China; ^3^Department of Medical Oncology, Xian Yang Central Hospital, Xianyang, China

**Keywords:** WDR5, MLL, histone H3K4 methylation, gene transcription, tumorigenesis, cancer therapy

## Abstract

The histone H3 lysine 4 (H3K4) presenter WDR5 forms protein complexes with H3K4 methyltransferases MLL1-MLL4 and binding partner proteins including RBBP5, ASH2L, and DPY30, and plays a key role in histone H3K4 trimethylation, chromatin remodeling, transcriptional activation of target genes, normal biology, and diseases such as MLL-rearranged leukemia. By forming protein complexes with other proteins such as Myc, WDR5 induces transcriptional activation of key oncogenes, tumor cell cycle progression, DNA replication, cell proliferation, survival, tumor initiation, progression, invasion, and metastasis of cancer of a variety of organ origins. Several small molecule MLL/WDR5 protein-protein interaction inhibitors, such as MM-401, MM-589, WDR5-0103, Piribedil, and OICR-9429, have been confirmed to reduce H3K4 trimethylation, oncogenic gene expression, cell cycle progression, cancer cell proliferation, survival and resistance to chemotherapy without general toxicity to normal cells. Derivatives of the MLL/WDR5 interaction inhibitors with improved pharmacokinetic properties and *in vivo* bioavailability are expected to have the potential to be trialed in cancer patients.

## Introduction

In eukaryotic cells, core histone proteins wrap around genomic DNA to form nucleosomes, the fundamental unit of eukaryotic chromatin structure (1, 2). Histone proteins are subject to a range of post-translational modifications, including acetylation, methylation, ubiquitination, sumoylation, hydroxylation, phosphorylation, and ADP-ribosylation.

Histone methylation mainly occurs at arginine (R) and lysine (K) residues located on histone H3 and H4 tails. While arginine residues are either mono- or di-methylated, histone lysine can be mono-(me1), di-(me2), or tri-(me3) methylated on the ε–amine group at diverse sites including H3K4, H3K9, H3K27, H3K36, H3K79, and H4K20 (3, 4).

Histone methylation plays different biological roles, depending on the histone and the residue modified, and are markers for transcriptional activation or repression. Histone H3K4 mono-methylation (H3K4me), di-methylation (H3K4me2), tri-methylation (H3K4me3), H3K9me, H3K27me, H3K36me3, H3K79me, and H3K79me2 are mainly involved in transcriptional activation (5–8). On the other hand, H3K9me2, H3K9me3, H3K27me2, H3K27me3, and H4K20me are markers for transcriptional repression (9, 10). The boundary elements that define the borders between euchromatin and heterochromatin protect against the spreading of H3K9 methylation heterochromatin signals into the neighboring H3K4 methylation euchromatin regions. On the other hand, the H3K9 methylation and corresponding heterochromatin-associated complexes prevent H3K4 methylation in the silent domain (11).

Histone H3K4 methylation is one of the most important histone modifications, and plays critical roles in regulating target gene transcription. Although commonly associated with transcriptional activation, histone H3K4me3 selectively localizes at gene promoters and transcriptional start sites and directly regulates gene transcription, whereas histone H3K4me1 and H3K4me2 are associated with enhancer elements and active genes respectively (12–14).

Histone H3K4 methylation is regulated by histone H3K4 demethylases, methyltransferases, and adaptors/presenters. H3K4 methylation is reversed by lysine-specific demethylase 1 (LSD1) (15) and four different jumonji/ARID domain-containing proteins (JARID1A-D) (16–19).

The mammalian H3K4 methyltransferases include SET domain containing 1A (SET1A)/lysine methyltransferase 2F (KMT2F), SET1B/KMT2G, mixed lineage leukemia 1 (MLL1/KMT2A, MLL2/KMT2B, MLL3/KMT2C, and MLL4/KMT2D proteins. The H3K4 methyltransferases alone show little catalytic activity. Their methyltransferase activities are enhanced only when the enzymes form complexes with their adaptor/presenter proteins, including retinoblastoma binding protein 5 (RBBP5), absent, small or homeotic-2 like (ASH2L), DPY30, and WD repeat domain 5 (WDR5) (14, 20–24), and further involved in gene transcription, pluripotency, self-renewal, and differentiation (20, 24–26). Among those methyltransferase adaptor proteins, WDR5 is of particular importance due to its unique protein structure, interaction with chromatin remodeling proteins, transcription factors and long non-coding RNAs, and its critical roles in gene transcription and tumorigenesis. Here we review our current understanding of how the H3K4me presenter WDR5 induces oncogene transcription and the initiation and progression of cancer of a variety of organ origins, and provide an overview of the anticancer effects and the mechanisms of action of small molecule MLL/WDR5 protein-protein interaction inhibitors.

## Structure and functions of WDR5 protein

### WDR5 protein structure

**Figure 1:**
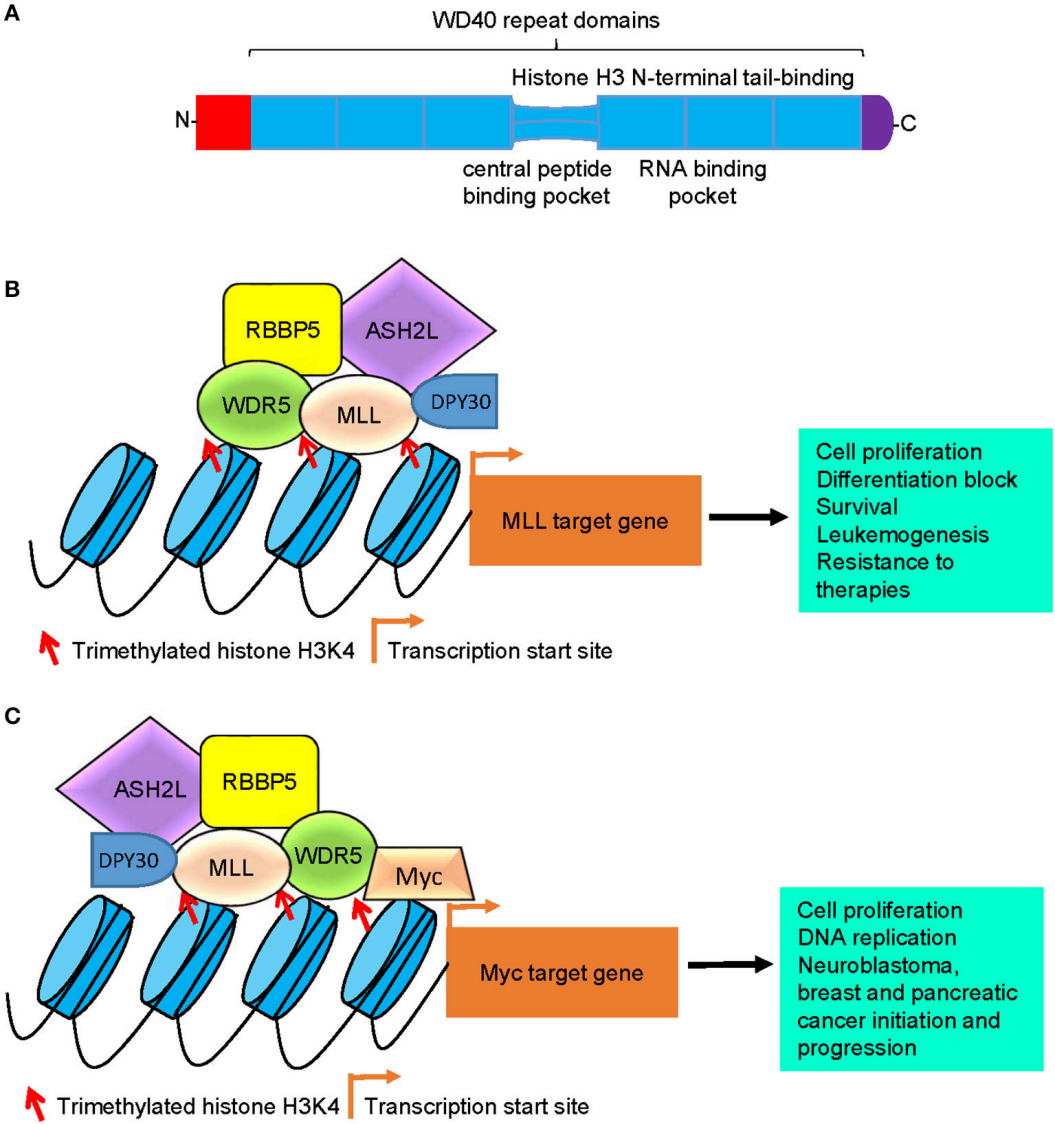
Models of WDR5-mediated histone H3K4 trimethylation, gene transcription, and tumorigenesis. **(A)** Schematic diagram of the WDR5 protein. The seven typical WD40 repeat domains interacts with the N-terminal end of the histone H3 tail, the central peptide-binding pocket interacts with the WIN motif of the SET domain of MLL histone methyltransferases, and the RNA-binding pocket between the 5th and 6th WD40 repeat domains is essential for WDR5 protein binding to lncRNAs. **(B)** WDR5 forms a protein complex with MLL, RBBP5, ASH2L, and DPY30 at MLL target gene promoters, leading to histone H3K4 trimethylation, MLL target gene transcription, cell proliferation, differentiation block, survival, leukemogenesis, and resistance to therapies. **(C)** WDR5 forms a protein complex with Myc, MLL, RBBP5, ASH2L, and DPY30 at Myc target gene promoters, leading to histone H3K4 trimethylation at Myc target gene promoters, Myc target gene transcription, cell proliferation, DNA replication, neuroblastoma, breast and pancreatic cancer initiation and progression. WDR5, WD repeat domain 5; MLL, mixed lineage leukemia 1; RBBP5, retinoblastoma binding protein 5; ASH2L, absent, small or homeotic-2 like; lncRNA, long non-coding RNA.

Structural studies reveal that the WDR5 protein consists of seven typical WD40 repeat domains, forming a seven-bladed propeller fold with each blade containing a four-stranded anti-parallel sheet, suggesting that WDR5 has many exposed surfaces (Figure 1A) (27). The WD40 domains are highly conserved structures that are involved in chromatin remodeling and gene transcription (28). The WD40 domains interact with the N-terminal end of the histone H3 tail, the central peptide-binding pocket of WDR5 protein interacts with the WIN motif of the SET domain of MLL H3K4 methyltransferase enzymes, and the WIN motif and the histone H3 tail compete for binding to WDR5 (29, 30) (Figure 1A). Due to its distinctive structure and many exposed surfaces, WDR5 also forms complexes with other proteins, such as the Cullin 4-DNA damage-binding protein 1 (CUL4-DDB1) ubiquitin ligase (31) and the transcription factors Pax7 (32), Oct4 (33), c-Myc (34), and N-Myc (35), and is therefore involved in a diverse range of biological processes (Table 1).

**Table 1:** The binding proteins, binding long non-coding RNAs and functions of the histone H3K4 methylation presenter WDR5.

**Binding proteins/RNAs**	**Functions**	**References**
**Proteins**	Modulating cell differentiation and inducing H3K4 trimethylation, gene transcription, pluripotency, tumorigenesis, and metastasis
SET1A-B, MLL1-4	Inducing histone H3K4 methylation and target gene transcription	(20, 24, 25, 36, 37)
CUL4-DDB1	Inducing histone H3K4 methylation and acting as an adaptor for CUL4-DDB1 ligase-mediated substrate recognition and proteolysis	(31)
Oct4	Inducing transcriptional activation of pluripotency genes	(33)
CHD8	Inducing HOXA2 gene transcription	(38)
GCN5	Modifying chromatin structure and regulating gene transcription	(39)
HDAC3	Increasing H3K4 trimethylation and mesenchymal gene expression	(40)
HDAC1, G9a, HDAC2, RERE	Regulating retinoic acid signaling and embryonic symmetry	(41)
MKL1	Enhancing MKL1-mediated pro-inflammatory gene transcription.	(42)
Pitx2	Inducing smooth muscle cell marker gene transcription and cell differentiation	(43)
Cbx8	Inducing Notch gene expression and tumorigenesis	(44)
N-Myc & c-Myc	Inducing Myc target gene transcription and tumorigenesis.	(34, 35)
TWIST1	Inducing HOXA9 gene transcription, prostate cancer cell migration, invasion and metastasis	(45)
**RNAs**	Inducing histone H3K4 trimethylation, gene transcription, stem cell renewal, cancer cell proliferation, lymphangiogenesis, and metastasis
RNA-binding	Essential for chromatin assembly, histone H3K4 trimethylation, gene transcription, and stem cell renewal	(46)
HOTTIP	Inducing histone H3K4 methylation at the HOXA gene locus and HOXA gene over-expression	(47)
NeST	Inducing H3K4 methylation and IFN-γ gene transcription, as well as susceptibility to viral and bacterial pathogens	(48)
Linc1405	Activating Mesp1 gene transcription	(49)
GClnc1	Inducing the transcription of oncogenes, such as SOD2, gastric cancer cell proliferation, invasion and metastasis	(50)
BLACAT2	Inducing VEGF-C expression, lymphangiogenesis and lymphatic metastasis	(51)

### WDR5 forms a protein complex with MLL, RBBP5, ASH2L, and DPY30 to facilitate histone H3K4 methylation

Also known as SWD3 and BIG-3, WDR5 is a core subunit of the human MLL1-4 histone H3K4 methyltransferase complexes. WDR5 and MLL proteins form core complexes with three other proteins: RBBP5, ASH2L, and DPY30 (Figure 1B; Table 1) (14, 20–23). Importantly, human MLL1-4 proteins alone show extremely low enzymatic activity, and their histone H3K4 trimethylation function is dramatically enhanced upon assembly of the protein complex (20). Whereas RBBP5, ASH2L, DPY30, and WDR5 all contribute to the optimal methylation effect of the MLL histone H3K4 methylation complexes, RBBP5, ASH2L, and DPY30 do not have stable interactions with MLL at the absence of WDR5, and the structural integrity of the H3K4 methylation complexes depends on WDR5 and MLL protein-protein interaction (20, 25, 36). Consistently, mutation of key WDR5 amino acid residues which disrupts WDR5 and MLL protein-protein interaction results in dissociation of the H3K4 methylation MLL complexes and blockage of H3K4 methylation (20, 37).

### WDR5 plays important roles in chromatin remodeling

The chromatin remodeling enzyme CHD8 interacts directly with WDR5, and depletion of CHD8 results in the loss of the WDR5/ASH2L/RBBP5 protein subcomplex and H3K4 trimethylation at the promoter of MLL target genes such as HOXA2 (Table 1) (38). WDR5 binds to the Polycomb protein Cbx8 to maintain histone H3K4 trimethylation on Notch-network gene promoters, so as to regulate Notch gene expression and mammary tumorigenesis (44). GCN5 histone acetyltransferase regulates diverse biological processes by inducing histone and non-histone protein acetylation. WDR5 forms a protein complex with Ada Two-A containing (ATAC)-type proteins and GCN5 to modify chromatin structure and regulate gene transcription (39). Under hypoxia, WDR5 gene expression is up-regulated, and up-regulated WDR5 interacts with the histone deacetylase HDAC3 to increase histone H3K4 trimethylation and mesenchymal gene expression (40). Additionally, WDR5 forms a protein complex with HDAC1, HDAC2, arginine-glutamic acid dipeptide repeats (RERE) protein and histone methyltransferase G9a to regulate retinoic acid signaling and embryonic symmetry (41).

### WDR5 forms protein complexes with transcription factors to regulate gene transcription

WDR5 directly interacts with the transcription factor MKL1 and the chromatin remodeling protein BRG1, and thereby enhances MKL1-mediated promoter activities of pro-inflammatory genes. Conversely, WDR5 knockdown blocks H3K4 trimethylation at the pro-inflammatory gene promoters and attenuates pro-inflammatory mediator production in macrophages (Table 1) (42). In embryonic stem cells, WDR5 gene expression positively correlates with the undifferentiated state, and is required for the efficient production of induced pluripotent stem cells and for the maintenance of embryonic stem cell self-renewal (33). Mechanistically, WDR5 interacts with the pluripotency transcription factor Oct4, and the interaction induces histone H3K4 trimethylation at the promoter regions of pluripotency genes and induces the transcription of the pluripotency genes, such as *Nanog, Sox2, and Myc*(33). In addition, WDR5 interacts with the transcription factor pituitary homeobox 2 (Pitx2) at the promoters of smooth muscle cell marker genes to induce histone H3K4 methylation, gene transcription and smooth muscle cell differentiation (43).

### WDR5 facilitates long non-coding RNA-mediated gene transcription

Mutation of key amino acid residues at the RNA binding pocket of WDR5 protein selectively abrogates its RNA binding without disrupting MLL/WDR5 protein complex assembly, but interrupts chromatin assembly, histone H3K4 trimethylation, gene transcription, and stem cell renewal (Table 1) (46). WDR5 has recently been shown to bind to long non-coding RNAs (lncRNAs), such as HOTTIP and NeST, to facilitate lncRNA-mediated histone H3K4 trimethylation and gene transcription (47–49, 51). Chromosomal looping brings the lncRNA HOTTIP into close proximity to its target genes, and HOTTIP RNA directly binds WDR5 and thus targets MLL/WDR5 histone H3K4 methylation complexes to the HOXA gene locus, leading to H3K4 trimethylation and HOXA gene transcription (47). The enhancer-like lncRNA NeST is causal for all phenotypes conferred by the murine viral susceptibility locus Tmevp3 which contains the NeST gene. NeST RNA binds WDR5 to alter histone H3K4 methylation at the IFN-γ gene locus and IFN-γ gene transcription, as well as susceptibility to viral and bacterial pathogens (48). WDR5 physically interacts with the lncRNA linc1405, the transcription factor Eomes and the histone acetyltransferase GCN5 at the enhancer region of the Mesp1 gene and activates its transcription during embryonic stem cell specification (49). The lncRNA BLACAT2 is markedly upregulated in metastatic bladder cancer cells, and BLACAT2 upregulates vascular endothelial growth factor C (VEGF-C) expression by binding to WDR5, resulting in bladder cancer-associated lymphangiogenesis and lymphatic metastasis (51).

## The role of WDR5 in cancer

### WDR5 promotes cancer initiation and progression

WDR5 has been well-established as an oncogenic factor for MLL-rearranged leukemia (52). Through interaction with its C-terminal, WDR5 recruits MLL to regulatory enhancers that are enriched for binding sites for E-twenty-six (ETS) family transcription factors, leading to the activation of leukemogenic genes and leukemia (53). The CCAAT/enhancer binding protein α (CEBPA) gene is mutated in 9% of acute myeloid leukemia samples, and C/EBPα p30 is the most common type of CEBPα mutation. WDR5 interacts with C/EBPα p30 at genomic regions enriched of histone H3K4 trimethylation marks, and is essential for C/EBPα p30-dependent leukemia cell self-renewal, myeloid differentiation block, and leukemogenesis (54).

Recent studies have revealed that WDR5 plays key roles in the tumorigenesis and progression of a variety of cancers (34, 35, 44, 55–60). Myc family oncoproteins are among the most prevalent oncogenic transcription factors, and c-Myc and N-Myc induce tumorigenesis by regulating gene transcription in human cancer tissues of various organ origins (34, 35, 55). WDR5 binds c-Myc and N-Myc proteins via their MYC box IIIb motif, and the interaction is essential for histone H3K4 trimethylation at Myc-responsive element E-Boxes of Myc target gene promotes, Myc target gene transcription and Myc-induced cancers, such as breast cancer and neuroblastoma (Figure 1C) (34, 35). Through screening patient-derived pancreatic ductal adenocarcinoma xenografts or genetically engineered mouse model-derived allografts, WDR5 has been found to be a top tumor maintenance gene for, and considerably over-expressed in, human pancreatic ductal adenocarcinomas (55). Mechanistically, WDR5 interacts with c-Myc to sustain proper DNA replication, and is essential for pancreatic ductal adenocarcinoma cell proliferation, tumor initiation, and progression (Figure 1C) (55).

The androgen receptor is a key factor in the progression of prostate cancer to castration-resistance. Upon androgen stimulation, PKN1 induces histone H3 threonine 11 phosphorylation (H3T11P) and WDR5 chromatin association at androgen receptor target gene loci (56, 57). WDR5 interacts with H3T11P, enhances MLL recruitment and histone H3K4 trimethylation at androgen receptor target gene loci, and consequently induces androgen receptor target gene transcription (56, 57). WDR5 is over-expressed in human prostate cancer tissues, induces prostate cancer cell proliferation, and drives castration resistance in prostate cancer cells (56, 57).

In a loss-of-function screen targeting 60 epigenetic regulators, the Polycomb protein Cbx8 has been found to be a key regulator of mammary carcinoma both *in vitro* and *in vivo* (44). WDR5 associates with Cbx8, maintains histone H3K4 trimethylation at Notch-network gene promoters and is required for Notch signaling activation and breast cancer tumorigenesis (44).

WDR5 is upregulated in bladder cancer tissues, and high levels of WDR5 expression positively correlate with advanced disease stage and poor patient survival (58). Mechanistically, WDR5 up-regulates cyclin B1, cyclin E1, cyclin E2, UHMK1, MCL1, BIRC3, and Nanog gene transcription through inducing histone H3K4 trimethylation, resulting in bladder cancer cell proliferation, self-renewal and chemo-resistance to cisplatin *in vitro* and tumor growth *in vivo* (58).

WDR5 is over-expressed in gastric and colon cancer cell lines and human tumor tissues, and high levels of WDR5 expression in tumor tissues is associated with poor patient survival rate (59, 60). Mechanistically, in gastric cancer cells, WDR5 induces histone H3K4 trimethylation at the Cyclin D1 gene promoter and Cyclin D1 gene transcription, leading to cancer cell proliferation (59). In colon cancer cells, WDR5 decreases the phosphorylation of the histone protein H2AX and induces H3K4 trimethylation, leading to colon cancer cell proliferation and survival, and WDR5 depletion sensitizes colon cancer cells to radiation-induced DNA damage (60).

### WDR5 induces cancer cell invasion and metastasis

Epithelial-mesenchymal transition is a key step in cancer cell invasion and metastasis. Under hypoxic conditions, WDR5 interacts with HDAC3 to activate mesenchymal gene transcription and to initiate hypoxia-induced epithelial-mesenchymal transition and metastatic phenotypes in non-small cell lung carcinoma cells (40). In lung squamous cell carcinoma and breast carcinoma cells, protein arginine methyltransferase 5 (PRMT5) complexes with MEP50/WDR77, and WDR5 is recruited by the protein complex to target gene promoters, resulting in histone H3K4 trimethylation, target gene transcription and cancer cell invasion (61). In addition, TWIST1 and HOXA9 are enriched in primary human prostate cancer tissues and even further over-expressed in metastatic tissues. By forming a protein complex with TWIST1, WDR5 induces HOXA9 gene transcription, prostate cancer cell migration, invasion, and metastasis (45).

### WDR5 induces lymphangiogenesis and lymphatic metastasis

High levels of the lncRNA BLACAT2 in human bladder cancer tissues is associated with lymphangiogenesis and lymphatic metastasis. WDR5 directly interacts with BLACAT2 to upregulate the expression of the critical lymphangiogenesis factor VEGF-C, leading to lymphangiogenesis and lymphatic metastasis (51). The lncRNA GClnc1 is up-regulated in human gastric cancer tissues, and WDR5 binds GClnc1 to regulate the transcription of oncogenes, such as SOD2, and consequently induces gastric cancer cell proliferation, invasion and metastasis (50).

## WDR5 inhibitors as anticancer agents

**Figure 2:**
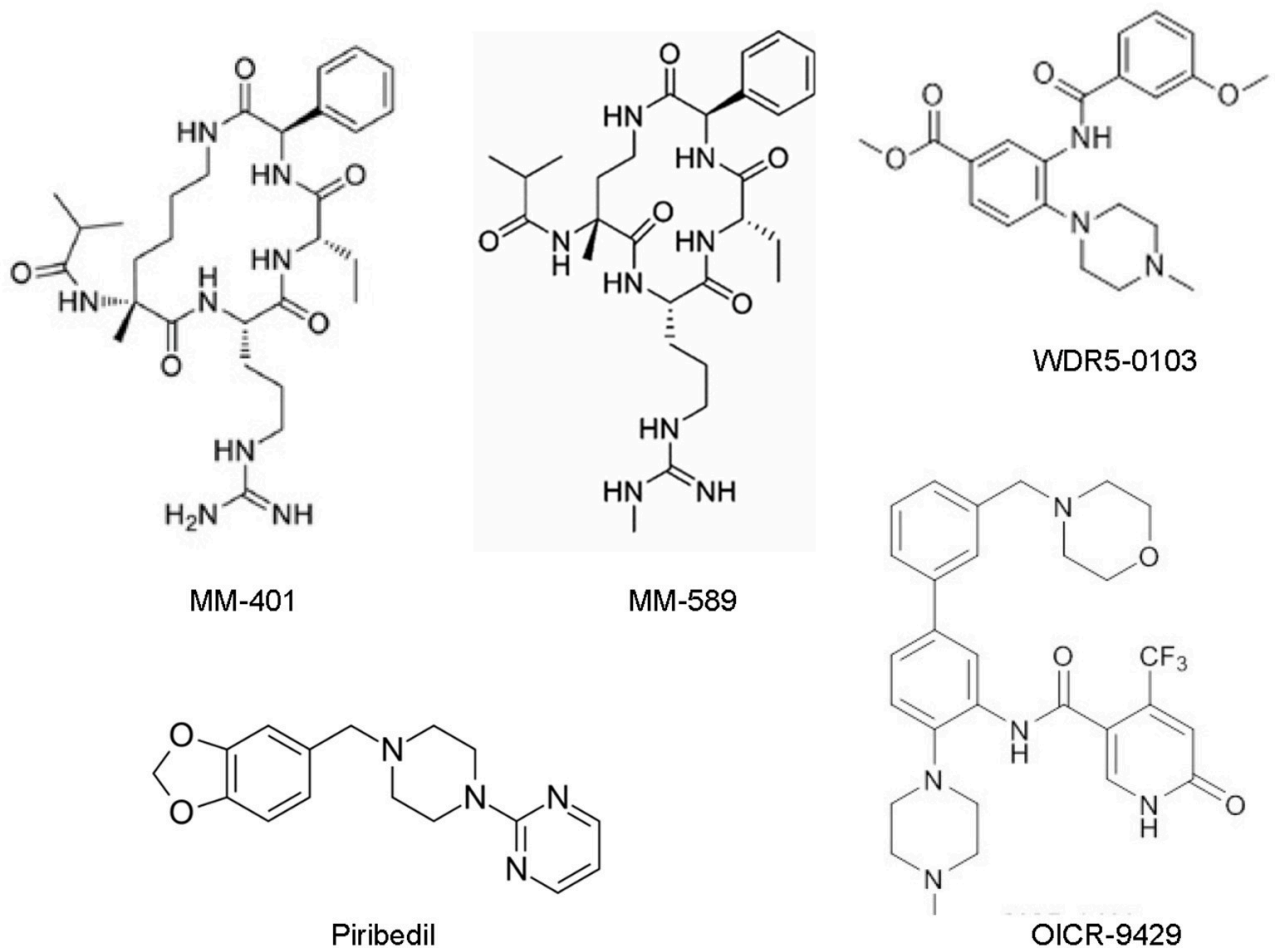
Chemical structures of the small molecular MLL/WDR5 protein-protein interaction inhibitors: MM-401, MM-589, WDR5-0103, Piribedil and OICR-9429. MLL, mixed lineage leukemia 1; WDR5, WD repeat domain 5.

**Figure 3:**
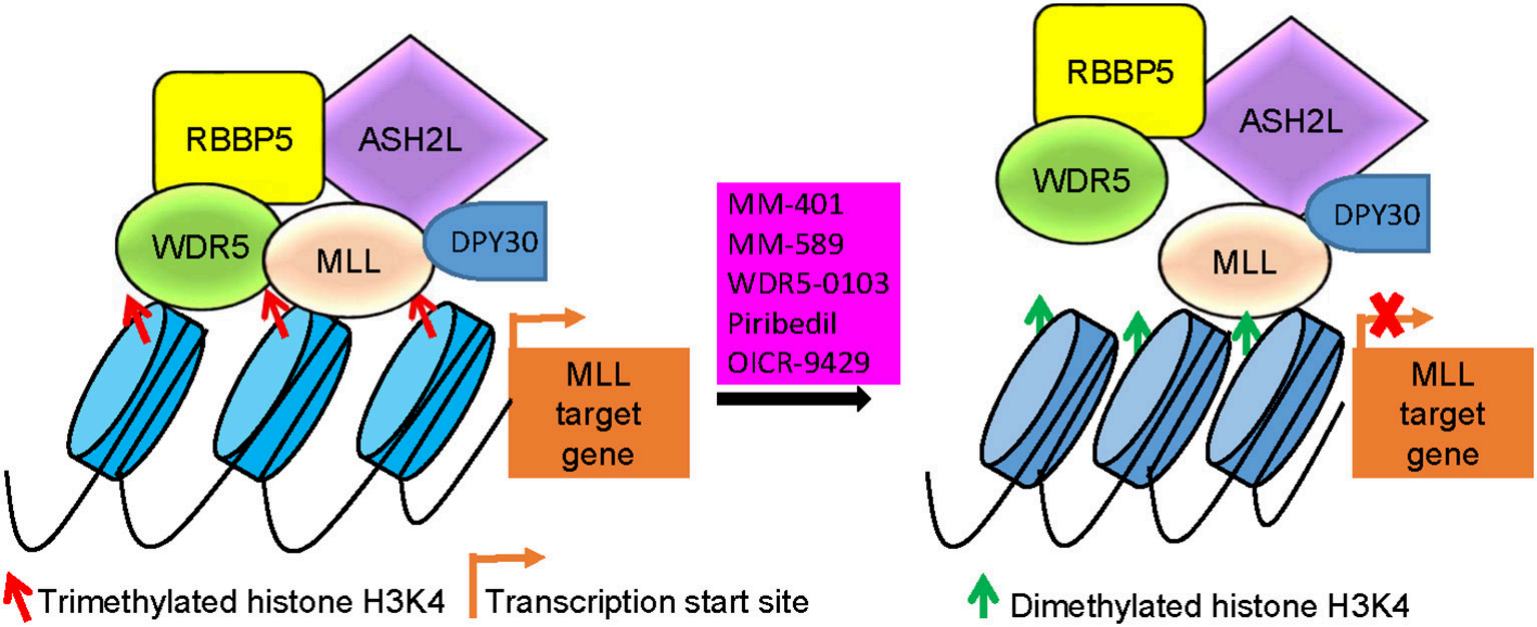
MLL/WDR5 protein-protein interaction inhibitors block MLL and WDR5 target gene transcription. MLL/WDR5 protein-protein interaction inhibitors disrupt MLL/WDR5/RBBP5/ASH2L/DPY30 protein complex formation at MLL target gene promoters, leading to the loss of histone H3K4 trimethylation and suppression of MLL target gene transcription. MLL, mixed lineage leukemia 1; WDR5, WD repeat domain 5; RBBP5, retinoblastoma binding protein 5; ASH2L, absent, small or homeotic-2 like.

WDR5 inhibitors are emerging as novel anticancer agents, and have been developed to prevent MLL and WDR5 protein-protein interaction and consequent oncogenic gene transcription (Figure 2; Table 2). Based upon -CO-ARA-NH-, the minimum binding motif derived from MLL, a large number of peptidomimetics have been designed to suppress MLL1-WDR5 interaction. One of such peptidomimetic, MM-102, effectively decreases the expression of HOXA9 and Meis-1, two critical MLL/WDR5 target genes in MLL fusion protein-driven leukemogenesis, and specifically induces growth inhibition and apoptosis in leukemia cells harboring MLL fusion proteins (62). The MLL/WDR5 protein-protein interaction inhibitor MM-401, compared with its enantiomer control MM-NC-401, suppresses histone H3K4 methyltransferase activity and reduces oncogenic gene transcription, resulting in MLL-rearranged leukemia cell growth inhibition, cell cycle arrest, myeloid differentiation and apoptosis without general toxicity to normal cells (Figures 2, 3; Table 2) (36). The macrocyclic peptidomimetic MM-589, on the other hand, binds WDR5 with a half maximal inhibitory concentration (IC_50_) of 0.90 nM (K_i_ value < 1 nM) and inhibits MLL/WDR5-mediated histone H3K4 methylation with an IC_50_ value of 12.7 nM. MM-589 potently and selectively inhibits MLL-rearranged leukemia cell proliferation and is >40 times more powerful than MM-401 (Figures 2, 3; Table 2) (63). The interaction between WDR5 and MLL catalytic domain peptides can also be antagonized with the small molecule compound WDR5-0103 which binds in the WDR5 peptide-binding pocket with a dissociation constant of 450 nM and inhibits histone H3K4 methylation activity of the MLL/WDR protein complex (Figures 2, 3; Table 2) (64).

**Table 2:** The anticancer effects of MLL/WDR5 protein-protein interaction inhibitors.

**Inhibitors**	**Known functions**	**References**
MM-102	Reducing HOXA9 and Meis-1 leukemogenic gene expression, and inducing MLL-rearranged leukemia cell growth inhibition and apoptosis	(62)
MM-401	Reducing oncogenic gene transcription and inducing MLL-rearranged leukemia cell growth inhibition, myeloid differentiation and apoptosis	(36)
MM-589	Potently and selectively inhibiting MLL-rearranged leukemia cell proliferation (>40 times more powerful than MM-401)	(63)
WDR5-0103	Reducing histone H3K4 methylation activity of the MLL/WDR5 protein complex	(64)
Piribedil	Inducing MLL-rearranged leukemia cell cycle arrest, growth inhibition, myeloid differentiation, apoptosis, and sensitivity to the chemotherapy	(65)
OICR-9429	Suppressing proliferation and inducing differentiation in C/EBPα p30 mutant acute myeloid leukemia cells, and blocking WDR5-N-Myc/c-Myc protein complex formation, Myc target gene expression and neuroblastoma and pancreatic ductal adenocarcinoma cell proliferation	(35, 54, 55)

Zhang et al. have recently screened a library consisting of 592 Food and Drug Authority-approved drugs for MLL inhibitors. Piribedil has been found to block the MLL/WDR5 interaction and thus to selectively reduce histone H3K4 methylation at MLL target gene loci and MLL target gene expression (Figures 2, 3). Piribedil specifically induces MLL-rearranged leukemia cell cycle arrest, growth inhibition, myeloid differentiation and apoptosis with little toxicity to non-MLL-rearranged cells, and sensitizes MLL-rearranged leukemia cells to doxorubicin-induced apoptosis (Table 2) (65).

The small molecule compound OICR-9429 binds the central peptide-binding pocket of WDR5, and competitively disrupts its interaction with of MLL with a high-affinity (Figure 2) (54, 66). OICR-9429 have shown anticancer efficacy against non-MLL-rearranged leukemia and solid tumors, through potently suppressing histone H3K4 trimethylation (Figure 3; Table 2) (35, 54, 55). The C/EBPα p30 mutant protein drives acute myeloid leukemia by forming a protein complex with WDR5. While showing no effect on C/EBPα p30-WDR5 protein-protein interaction, OICR-9429 disrupts MLL/WDR5 complex formation, selectively upregulates myeloid-specific transcripts, suppresses proliferation and induces differentiation in C/EBPα p30 mutant human acute myeloid leukemia cells (Table 2) (54). In neuroblastoma cells, OICR-9429 blocks WDR5-N-Myc protein complex formation, reduces N-Myc target gene expression, and induces N-Myc gene-amplified neuroblastoma cell growth inhibition (35). In pancreatic ductal adenocarcinoma cells, OICR-9429 blocks WDR5-c-Myc protein complex formation and effectively reduces tumor cell clonogenic capacity (55).

## Conclusions and perspective

Histone H3K4 methylation plays a major role in regulating the expression of genes important for pluripotency, stemness, development, and diseases including cancer. Through forming protein complexes with H3K4 methyltransferases including SET1A/B and MLL1-4 as well as adaptor/presenter proteins including RBBP5, ASH2L, and DPY30, WDR5 plays an essential role for histone H3K4 trimethylation, transcriptional activation of target genes, normal biology, and diseases such as MLL-rearranged leukemia. By forming protein complexes with a number of other oncoproteins, such as c-Myc and N-Myc, WDR5 plays a key role in transcriptional activation of oncogenes essential for cell cycle progression, DNA replication, cell proliferation, survival, tumorigenesis, progression, invasion and metastasis of cancer of a variety of organ origins. Future research should be performed to identify novel WDR5 binding proteins, through unbiased protein immunoprecipitation and mass spectrometry analysis, in cancer cells of various organ origins, so as to discover novel oncogenic pathways and therapeutic targets.

The unique structural feature of MLL/WDR5 protein-protein interaction has made it an emerging drug target. Several small molecule compounds have been developed to suppress the interaction between WDR5 and MLL catalytic domain, such as MM-401, MM-589, WDR5-0103, Piribedil, and OICR-9429. Treatment with the small molecule MLL/WDR5 interaction inhibitors results in reduced H3K4 trimethylation, transcriptional suppression of oncogenic gene expression, tumor cell growth inhibition, cell cycle arrest, differentiation, and apoptosis without general toxicity to normal cells. Nevertheless, none of the inhibitors has been tested in mouse models of cancer. Future synthesis of or screening for more potent MLL/WDR5 protein-protein interaction inhibiting compounds with ideal pharmacokinetics and bioavailability *in vivo*, is likely to provide drug-like MLL/WDR5 protein-protein interaction inhibitors suitable for clinical trials in cancer patients.

## Author contributions

KL and QC conceived and designed the review. KL, HT, XS, and QC wrote the manuscript and the figures. All authors read and approved the final manuscript.

### Conflict of interest statement

The authors declare that the research was conducted in the absence of any commercial or financial relationships that could be construed as a potential conflict of interest.
